# Holographic inspired high-performance circular polarized spoof surface plasmon polariton leaky-wave antenna excited by a novel launcher

**DOI:** 10.1038/s41598-025-85300-y

**Published:** 2025-01-07

**Authors:** Sajjad Zohrevand, Mohammad Amin Chaychi Zadeh, Ehsan Farokhipour, Daniel Erni, Nader Komjani

**Affiliations:** 1 Department of Electrical Engineering, Iran University of Science and Technology, Tehran, 16846-1314 Iran; 2 General and Theoretical Electrical Engineering (ATE), University of Duisburg-Essen, and CENIDE-Center for Nano Integration Duisburg-Essen, 47048 Duisburg, Germany

**Keywords:** Electrical and electronic engineering, Engineering

## Abstract

The holographic technique is one of the simplest methods for designing antennas based on metasurface. This paper presents a spoof surface plasmon polariton (SSPP) leaky-wave antenna (LWA) based on the concept of impedance modulated metasurfaces by the anisotropic holographic technique. Instead of parasitic elements, anisotropic SSPP elements are exploited to achieve radiation with circular polarization. The characteristics of the SSPP elements are obtained by the aperture field estimate method. The hologram surface consists of hollow cross-bars unit cells. The anisotropy of each unit cell is achieved by combining the transformation optic method and the particle swarm optimization algorithm. A major challenge of the SSPP LWA based on modulated impedance surfaces is to find a suitable excitation technique. This study proposes a waveguide strip line launcher for excitation to minimize interference on the radiation pattern beam. The designed launcher provides a good impedance matching from 8 to 20 GHz, with an impedance bandwidth of 142%. The peak gain, radiation efficiency, axial ratio (AR) bandwidth, and side lobe level at the design frequency of 18 GHz are 19.7dBi, 93%, 11%, and − 12.1 dB, respectively. After optimizations and simulations are conducted using MATLAB and CST software, the proposed antenna is fabricated, and its radiation characteristics are measured. The measured results agree well with the simulated ones, indicating the high validity of the method.

## Introduction

In modern point-to-point communication systems, antennas with high directivity and gain are highly demanded. Leaky-wave antennas (LWAs) meet this requirement due to their large dimensions compared to the wavelength. Researchers are always interested in LWAs due to their unique features. These antennas require no complex feeding network and minimize the influence of the feeding network on the radiation pattern beam. Also, the field amplitude distribution in the antenna aperture can control the side lobe level (SLL) in LWAs. In addition, it is possible to scan the space by changing the frequency without using additional mechanical equipment, which is a special feature in radar applications^1–5^. Generally, LWAs are divided into uniform (fast wave) and periodic (slow wave) structures. Uniform LWAs often scan a quarter of the front space and cannot operate near the broadside. However, periodic LWAs can scan from backward to forward through broadside direction, depending on the Floquet harmonic order and their operating frequency^6,7^.

Due to the coupling of photons and free electrons of the metal, a particular kind of surface electromagnetic wave exists in the optical frequency range called surface plasmon polariton (SPP)^7^. The SPPs can strongly confine light in the sub-wavelength scale. Also, SPPs have found many applications owning to their ability to surpass the diffraction limit. However, since metals behave like perfect electrical conductors (PECs) at lower frequencies (microwave and terahertz range), natural SPPs are unavailable at the metal–dielectric interface^8–10^. To overcome this limitation, spoof-surface plasmonics have been proposed to mimic SPPs properties in microwave and terahertz ranges. Spoof surface plasmon polariton (SSPP) waves have been widely studied in recent years^11–15^. These waves are density longitudinal fluctuations of surface charges propagated at the boundary between dielectric and metal without radiation loss. Since a flat boundary between a metal and a dielectric can only be excited by TM-polarized wave radiation, this wave can be coupled to a surface wave. The reason for this event can be seen in the need for the vertical component of the electric field to create a surface-polarized charge. Waves with p or TM polarization have an electric field component in the radiation plane. In contrast, waves with s or TE polarization do not have this feature, and the electric field is perpendicular to the radiation plane^16,17^.

Depending on the interference concept established in the optical band, the holographic principle is used in the original version to record and reconstruct object image in real three-dimensional space. This technique can be used similarly to antenna design in lower frequency bands to characterize an antenna’s aperture electromagnetic properties in relation to both reference and object waves. In this method, the antenna feed’s electric field distribution at a hologram plane is regarded as the reference wave, whereas the intended radiation pattern corresponds to the object wave^18^. The holographic technique is a method to synthesize metasurface (2D metamaterial) antennas. Various design methods have been proposed for this purpose, such as the Huygens^19^, the phase gradient^20^, the dispersion diagram engineering^21^, etc. The holographic method synthesizes 2D metamaterial antennas using the concept of surface impedance. Some advantages of the holographic technique are its frequency independence, non-dependence of the reference and the object waveform, and its potential usage in coherent structures. Gradually, the holographic technique is used in designing antennas in different frequency bands due to its advantages, such as increasing gain and radiation efficiency, miniaturization of the structure, and reducing the radar cross section (RCS)^22–26^. To design holographic-based metaradiator, the reference wave is the spatial^26^ or surface wave in LWAs^27^. The object wave (radiation pattern) is completely independent of the reference wave and is determined arbitrarily.

This paper presents a CP LWA based on an anisotropic holographic technique and aperture field estimation (AFE) method. Although various solutions have been proposed to feed SSPP LWAs, we also propose a novel launcher as a feed network of the LWA. One of the advantages of the proposed launcher is the very low impact on the structure’s radiation pattern and the achievement of high gain and efficiency compared to similar structures without parasitic elements. The hollow cross-bars unit cell has been used to implement the SSPP LWA on Rogers 4003 substrate with a thickness of 0.524 mm at the design frequency of 18 GHz. In the following, a combination of the transformation optic (TO) relations and the particle swarm optimization (PSO) algorithm are used to find the corresponding dimensions and rotation angle of each hollow cross-bars of each unit cell with the components of the surface impedance tensor. A good agreement between the simulation and measurement results can be observed after measurements of the fabricated sample.

## Design process

### Antenna design based on holographic technique

**Fig. 1:**
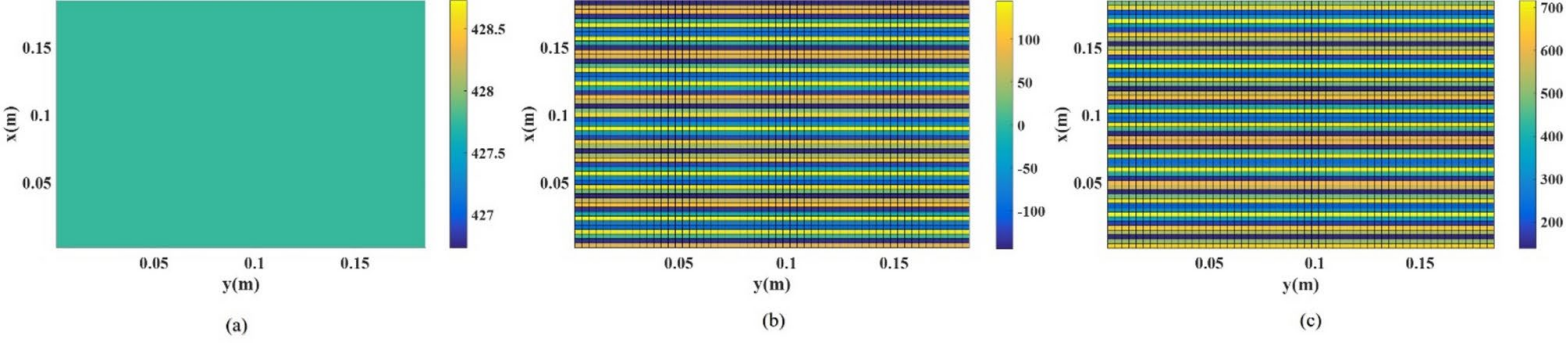
Distribution of the imaginary surface impedance matrix elements at 18 GHz (**a**) Z_xx_, (**b**) Z_xy_, and (**c**) Z_yy_.

The holographic technique is used to implement artificial surface impedances for the pattern synthesis of leaky-wave antennas, as shown conceptually in Fig. 1. Based on the holographic principle, an object wavefront can be recovered from a hologram, which has recorded the interference of object and reference waves^28^. In general, the impedance matrix components distribution relation of the tensor hologram and recorded waves can be written as follows^27^:

1 $$\overline{\overline{Z}}_{S} = j\left( {\begin{array}{*{20}l} X \hfill & 0 \hfill \\ 0 \hfill & X \hfill \\ \end{array} } \right) + j\frac{M}{2}{\text{Im}} \left\{ {\vec{\psi }_{obj} \otimes \vec{\psi }_{ref}^{\dag } - \vec{\psi }_{ref} \otimes \vec{\psi }_{obj}^{\dag } } \right\}$$

where *ψ*_*ref*_ and *ψ*_*obj*_ represent the reference and object waves, respectively. X is the average surface impedance, and M is the modulation coefficient of the isotropic unit cell. The values of the average surface impedance and the modulation coefficient are obtained from Eqs. (9) and (10), respectively. Also, “*†*” and “⊗” denote the Hermitian conjugate and outer product signatures, respectively. For a reference wave with a planar wavefront traveling in the y-direction with propagation constant of *k*, and an object wave radiated in the desired direction with circular polarization, reference and object waves at the hologram plane are written as:

2 $$\vec{\psi }_{ref} = (0,1,0)e^{ - jkny}$$

3 $$\vec{\psi }_{obj} = ( \pm j\cos \theta ,1, \mp j\sin \theta \cos \varphi )e^{ - jk(x\sin \theta \cos \varphi + y\sin \theta \sin \varphi )}$$

where *θ* and *ϕ* are the main lobe direction of the object beam in a spherical coordinate system. Positive and minus signs in (3) indicate RHCP and LHCP, respectively. *n* represents the effective refractive index of surface impedance and is obtained from $n=\sqrt{1+{X}^{2}}$^24^. According to Eqs. (1–3), the imaginary part of surface impedance matrix elements could be written as follows:

4 $$Z_{xx} = X$$

5 $$Z_{yy} = X + M\sin (kny - k\sin \theta (x\cos \varphi + y\sin \varphi ))$$

6 $$Z_{xy} = Z_{yx} = \pm \frac{M}{2}\cos \theta \cos (kny - k\sin \theta (x\cos \varphi + y\sin \varphi ))$$

Herein, the radiation direction of the two broadside object beams with RHCP and LHCP is considered (*ϕ* = π/2*, θ* = *0*, π) at 18 GHz with the same gain. Figure 1 illustrates the components Z_xx_, Z_yy_, and Z_xy_ of the interface pattern for the LWA structure, assuming *X* = 427.5 and *M* = 288.5, respectively [According to Eqs. (9) and (10)].

**Fig. 2:**
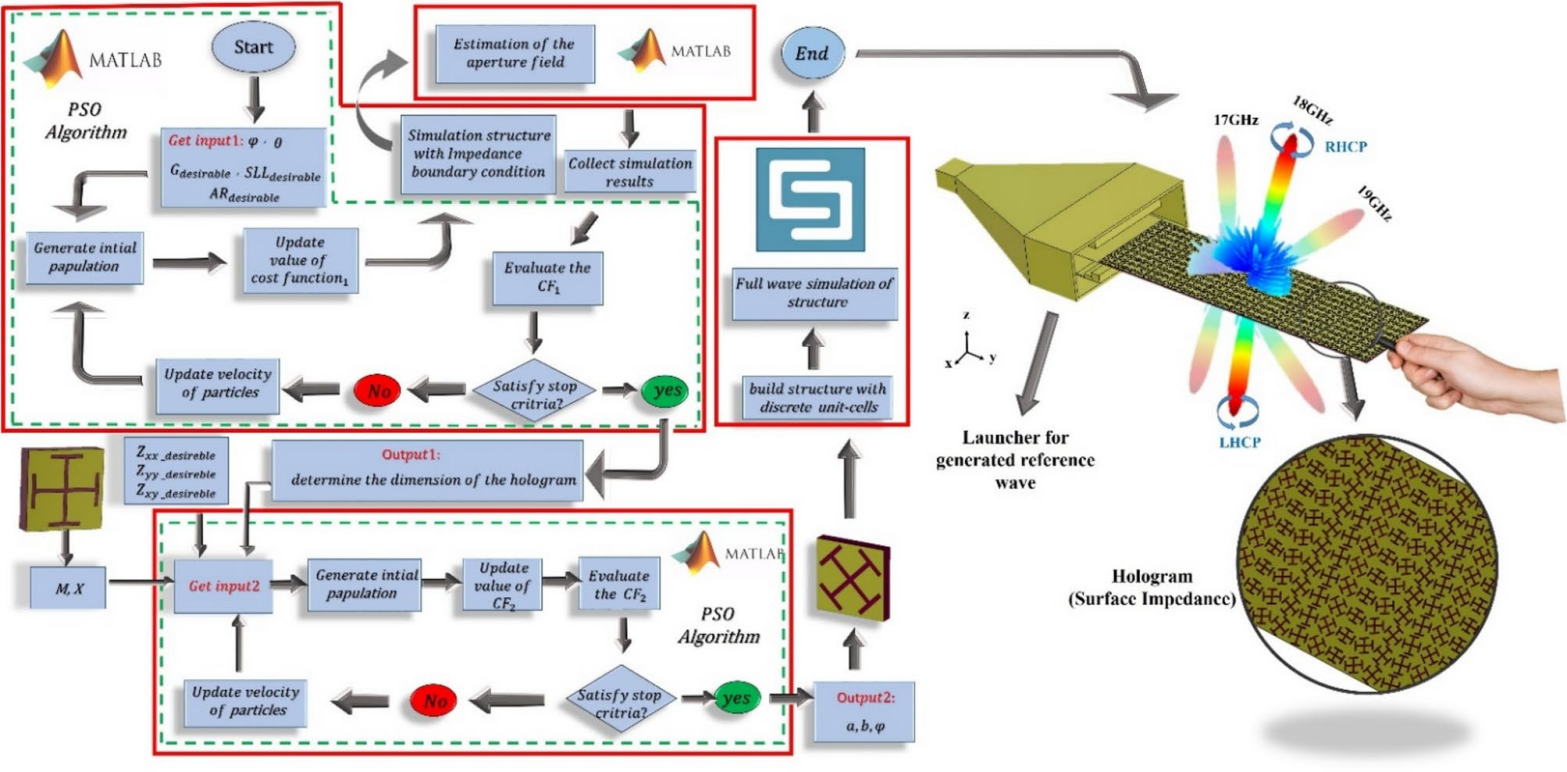
Schematic of the overall structure with a description of the design process.

The cost function of the radiation specifications of the holographic based LWA at the design frequency of 18 GHz is as follows:

7 $$CF_{1} = \omega_{1} \left[ {10 - \left| {SLL_{E - plane} } \right|} \right]^{2} + \omega_{2} \left[ {G_{desirable} - \left| {G^{{\left[ {dB} \right]}} } \right|} \right]^{2} + \omega_{3} \left[ {AR_{desirable} - \left| {AR^{{\left[ {dB} \right]}} } \right|} \right]^{2}$$

where $\omega_{1}$, $\omega_{2}$ and $\omega_{3}$ are the weight factors of each error function term. Figure 2 depicts the flowchart of the proposed LWA design, which minimizes the above cost function.

In step (I) of the flowchart, the length (*L*) and width (*w*) of the overall hologram plane are determined. Also, in step (II), the arm lengths (*a* and *b*) and rotation angle (*φ*) of each hologram unit cell are extracted. The details of each step of the design flowchart are described in detail in the following sections.

### Proposed unit cell to realize the desired hologram

**Fig. 3:**
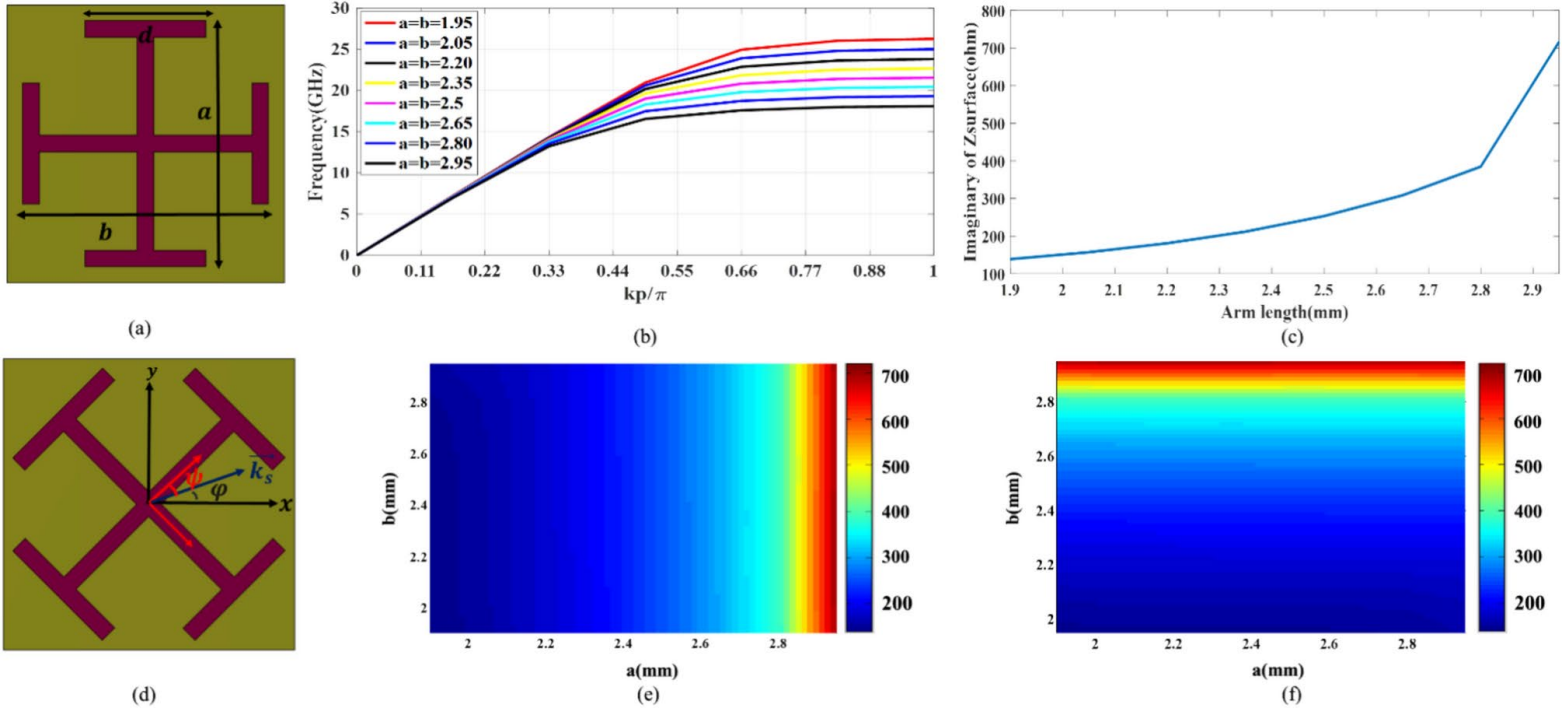
(**a**) The isotropic state of the proposed unit cell, (**b**) the dispersion diagram of the isotropic unit cell, (**c**) the surface impedance curve of the isotropic unit cell at 18 GHz, (**d**) the anisotropic state of the proposed unit cell, the variation of (**e**) *X*_1_, and (**f**) *X*_2_ in terms of small and large arm length of the cross-bars.

Obtaining the dispersion diagram of a periodic lattice made up of the hologram’s unit cells is a crucial step in defining the surface impedance of a hologram. Circular, square, and hexagonal patches unit cell or slots are best suited for implementing surface impedance^29^. The unit cell’s size should be sufficiently small comparing the operating effective wavelength. Moreover, the substrate thickness should be greater than one-tenth of the effective wavelength to promote surface wave excitation. The proposed unit cell in this paper, consists of hollow metallic cross-bars printed on a groundless Rogers 4003 substrate with a thickness of 0.524 mm, as shown in Fig. 3a. In the first step, the isotropic state of the unit cell was simulated in the Eigen-mode solver of CST software with the appropriate boundary conditions, whose dispersion diagrams are shown in Fig. 3b. The presence of a ground plane creates a hybrid mode of TEM and SSPP, and this article aims to excite the pure SSPP mode. Generally, there are three main approaches to determine the surface impedance of a scalar unit cell^29^. Here, we calculate the surface impedance using the dispersion curve and equation below^30^:

8 $$Z_{s} = \eta_{0} \sqrt {\left( {{\phi \mathord{\left/ {\vphantom {\phi {k_{0} p}}} \right. \kern-0pt} {k_{0} p}}} \right)^{2} - 1}$$

where *ϕ* is the phase difference within a unit cell along the propagation direction of the reference wave. Also, *p* is the period of the unit cells, and observing the Nyquist sampling rate is considered 3.33 mm, at the design frequency of 18 GHz. The values of physical parameters of *d* and *s* are considered 1.5 mm and 0.2 mm, respectively. Figure 3c illustrates the curve of variations of imaginary parts of surface impedance versus different lengths of arm length at the operation frequency of 18 GHz. It is evident that variation of the arm length (*a* = *b*) from 1.90 to 2.95 mm, leads to the impedance range coverage of 577 Ω from 139 to 716 Ω. The average surface impedance and the modulation coefficient could be calculated as:

9 $$X = \frac{1}{2}\left[ {Max\left( {\eta_{surf} } \right) + Min\left( {\eta_{surf} } \right)} \right]$$

10 $$M = \frac{1}{2}\left[ {Max\left( {\eta_{surf} } \right) - Min\left( {\eta_{surf} } \right)} \right]$$

Therefore, the values of *X* and *M* are equal to 427.5 and 288.5, respectively.

Figure 3d demonstrates the proposed anisotropic unit cell with a hollow cross-bars metallic patch. Three design criteria are taken into consideration for altering the impedance of the anisotropic unit cell: the cross-bars’ rotation angle ($\psi$), small (*a*), and large (*b*) cross-bars length. The following equations are used to compute the surface impedance matrix^31^:

11 $$Z_{xx} = \cos^{2} (\psi - \varphi )X_{1} + \sin^{2} (\psi - \varphi )X_{2}$$

12 $$Z_{yy} = \sin^{2} (\psi - \varphi )X_{1} - \cos^{2} (\psi - \varphi )X_{2}$$

13 $$Z_{xy} = \sin (\psi - \varphi )\cos (\psi - \varphi )(X_{1} - X_{2} )$$

where $\psi$ and $\varphi$ are illustrated in Fig. 3d. When the rotation angle is zero, *X*_1_ and *X*_2_ are the unit cell surface impedances aligned with the principal axes. Indicators of *X*_1_ and *X*_2_ are obtained such as the isotropic unit cell surface impedance calculation, using parametric sweeping simulation in the CST Eigenmode solver^32^. Figure 3e–f depicts variation of *X*_1_ and *X*_2_ in terms of variation in *a* and *b*.

### Estimation of the aperture field

The generalized version of the holographic technique is suggested when implementing an anisotropic hologram using the aperture field estimation (AFE). It is proposed that for the SSPP leaky-wave meta-radiator with a circularly-polarized (CP) pencil beam, the aperture field estimation is used to know the distribution of the near field of the object beam at the surface of the hologram aperture (${E}_{a}$), where ${\overrightarrow{E}}_{a}$ possesses the general form of

14 $$\vec{E}_{a} \left( {x,y} \right) = E_{ax} \left( {x,y} \right)\hat{x} + E_{ay} \left( {x,y} \right)\hat{y}$$

Generally, the far field radiation pattern relations of an antenna can be written as^33^:

15 $$\vec{E}_{F} (r,\theta ,\varphi ) \approx \frac{{jke^{ - jkr} }}{2\pi r}\left[ {F_{\theta } (\theta ,\varphi )\hat{\theta } + F_{\varphi } (\theta ,\varphi )\hat{\varphi }} \right]$$

16 $$F_{\theta } (\theta ,\varphi ) = f_{x} \cos \varphi + f_{y} \sin \varphi$$

17 $$F_{\varphi } (\theta ,\varphi ) = \cos \theta ( - f_{x} \sin \varphi + f_{y} \cos \varphi )$$

where ${f}_{x}$ and ${f}_{y}$ are the spectrum domain of the aperture field of ${E}_{ax}$ and ${E}_{ay}$ at the hologram plane respectively, and are expressed as

18 $$f_{x} (k_{x} ,k_{y} ) = \iint_{ap} {E_{ax} (x^{\prime},y^{\prime})e^{{j(k_{x} x^{\prime} + k_{y} y^{\prime})}} dx^{\prime}dy^{\prime}}$$

19 $$f_{y} (k_{x} ,k_{y} ) = \iint_{ap} {E_{ay} (x^{\prime},y^{\prime},z^{\prime})e^{{j(k_{x} x^{\prime} + k_{y} y^{\prime})}} dx^{\prime}dy^{\prime}}$$

${E}_{ax}$ and ${E}_{ay}$ in Eq. (14) may be selected arbitrarily. This demonstrates the simplicity of polarization manipulation by anisotropic structures. For instance, the prerequisite for circular polarization is:

20 $$F_{\varphi } (\theta_{0} ,\varphi_{0} ) = \pm jF_{\theta } (\theta_{0} ,\varphi_{0} )$$

which for radiation in the broadside direction leads to:

21 $$E_{ax} (x^{\prime},y^{\prime}) = \pm jE_{ay} (x^{\prime},y^{\prime})$$

**Fig. 4:**
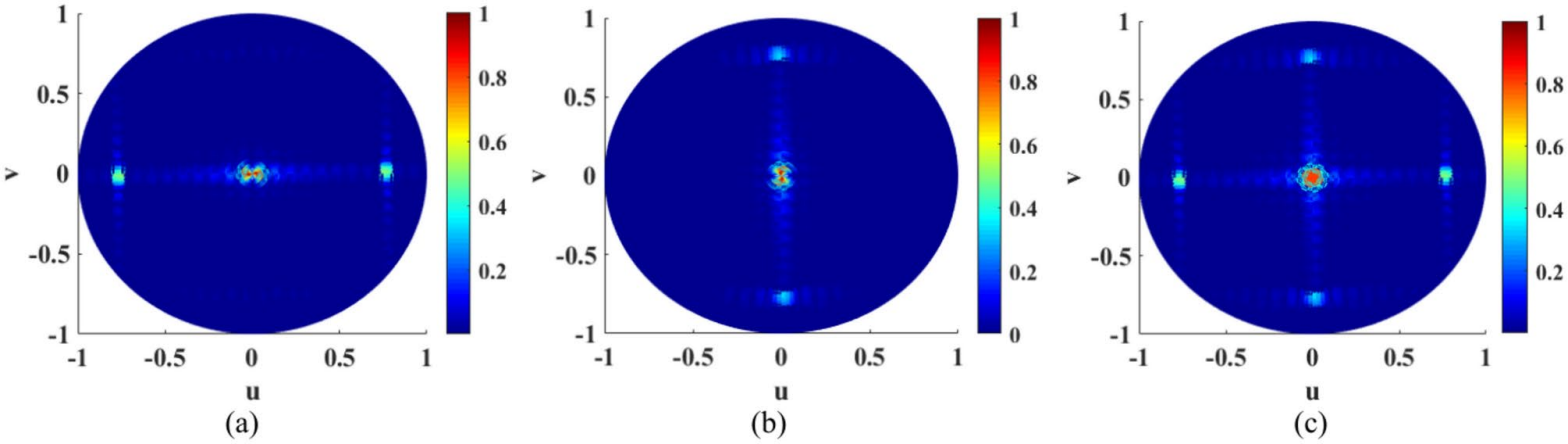
(**a**) Horizontal and (**b**) vertical components $\left( {F_{\theta } ,F_{\varphi } } \right)$ and (**c**) absolute of the normalized total far-field radiation pattern in the u–v plane for the meta-radiator with CP broadside radiation.

Therefore, the horizontal and vertical components $\left( {F_{\theta } ,F_{\varphi } } \right)$ and absolute of the normalized total far-field radiation pattern in the u-v plane for the meta-radiator with CP broadside radiation are illustrated in Fig. 4.

### Design of the launcher as the source of the reference wave

Various types of reference and object waves can be considered in holographic antennas because the nature of the holographic technique is not independent of the type and number of object and reference waves^34,35^. The reference wave is applied to the hologram surface through a launcher as a feed network, so the criteria of the reference wave depend on the launcher. In reflect array and transmit array antennas, the reference wave is in spatial form and it is generated by separate radiating structures like horn antennas and irradiated to the hologram interface plane. In leaky-wave holographic antennas, the reference wave is a surface wave. In the design of the surface wave launcher, several points should be considered: First, possess least cross polarization, second, create a surface wave with high polarization purity, third, achieve a good impedance, momentum and modal matching with the holographic plane, fourth, relieve least destructive effect on the far field radiation pattern^36^.

**Fig. 5:**
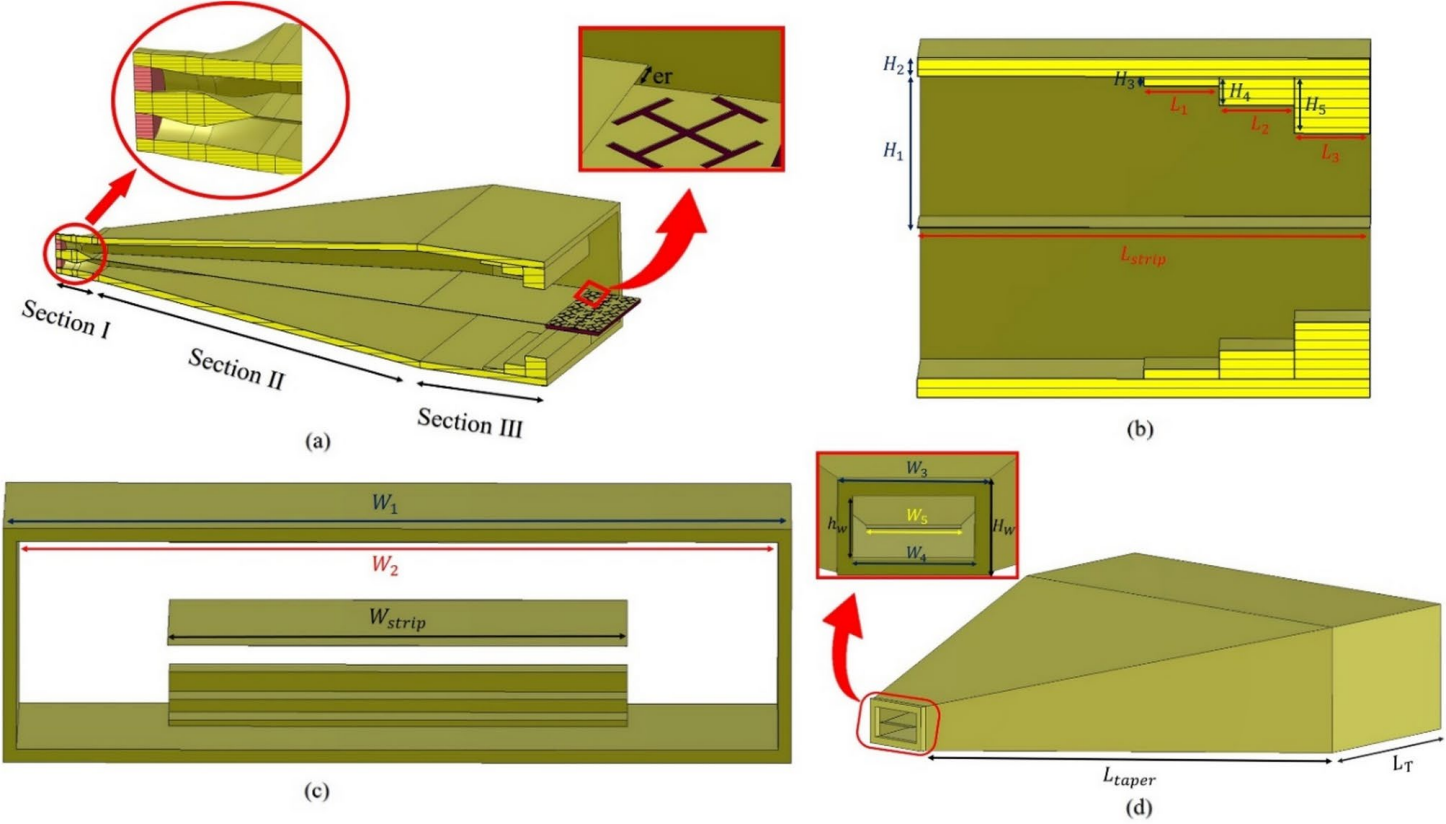
Schematic of the proposed launcher (**a**) different parts, (**b**) side view, (**c**) front view, and (**d**) zoom-in of transition part for connection to coax.

Meta-radiators based on the leaky-wave mechanism can be fed from the edge or inside of the structure. Various launchers have been used for leaky-wave antennas based on sinusoidal modulated surface impedance^37,38^. The grounded unit cells support the TM dominant mode. The dominant mode of ungrounded unit cells, whether electrically connected or not, is TM and TE, respectively. As mentioned, the ungrounded holographic structure with modulated surface impedance can support the propagation of a SSPP wave with a dominant TM_0_ mode. In other words, we need to design a launcher capable of producing a TM_0_ mode without a ground plane to support SSPP modes^39–42^. Up to now, launchers like microstrip^43^, SIW^44^, slotline^45^, etc., have been utilized to excite SSPP modes. This section aims to design a launcher that excites SSPP modes, and has a little destructive effect on the radiation pattern. Figure 5a shows three sections of the proposed transition between the strip line and the SSPP elements. Section I includes a coax cable with a characteristic impedance of 50 Ω, which is matched to a strip-line with a line width of 1 mm by changing the thickness and shape of the coax cable. In section II, a tapered strip-line matches two strip-lines with different line widths. In section III, stepped impedance efficiently transfers the surface wave from the strip-line to the SSPP elements with simultaneous impedance, modal, and momentum matching. The impedance of section I is 50Ω. Section II changes the impedance from 50 to 35.65 Ω. In Section III, the specific impedance is changed by the impedance step structure according to Table 1. Figure 5b,c show the side and front view of the launcher respectively, where stepped impedance is located. Also, Fig. 5d shows the external view of the proposed launcher, the zoomed part in Fig. 5d is where the strip line and coaxial cable will be connected.

**Table 1:** The characteristic impedance of each section in the proposed launcher.

Section I	Section II	Section III		
		Impedance step structure with height H_3_	Impedance step structure with height H_4_	Impedance step structure with height H_5_
50 Ω	From 50 to 35.65 Ω	33.92 Ω	30.20 Ω	24.08 Ω

**Fig. 6:**
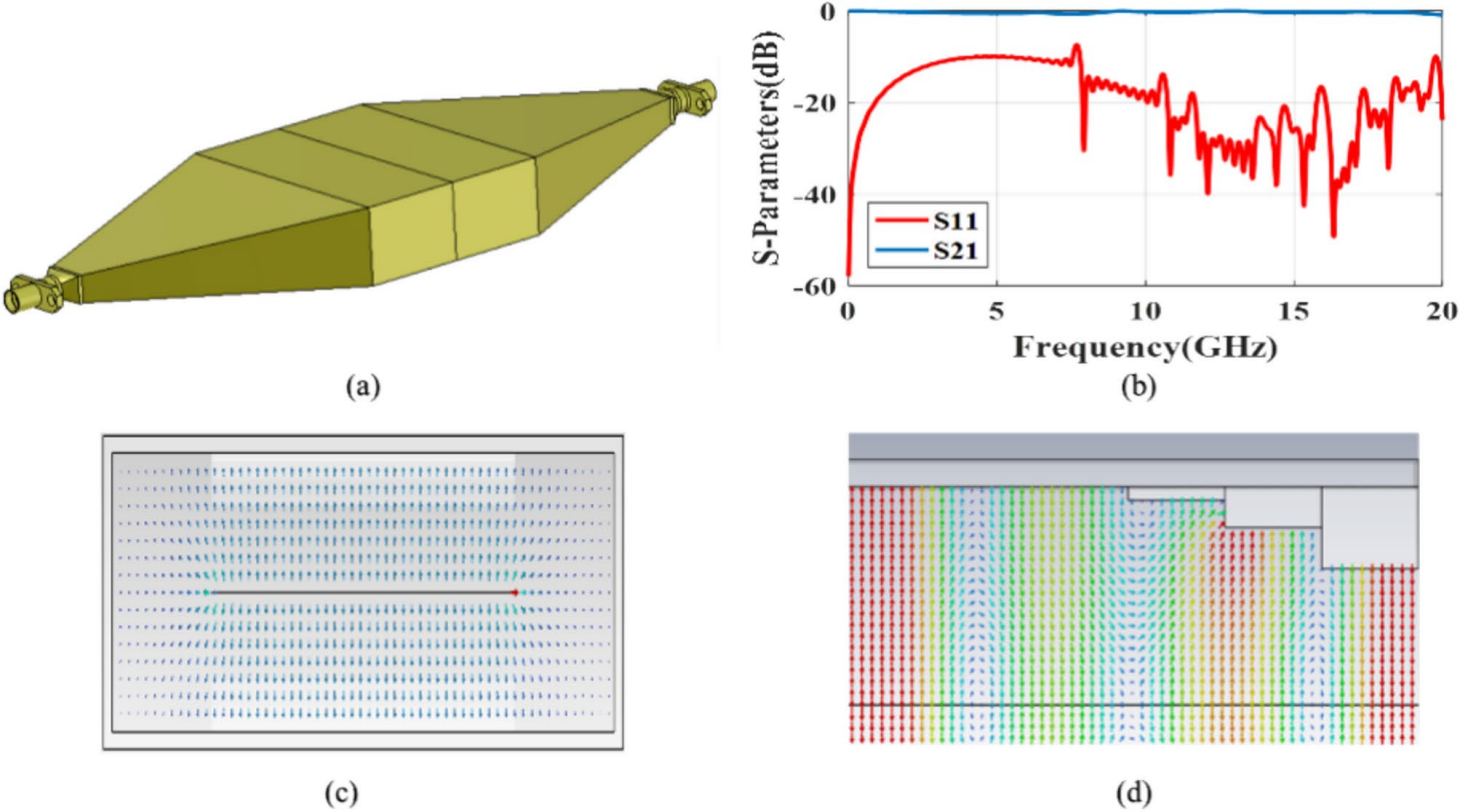
(**a**) Back-to-back connection of the proposed launcher, (**b**) simulated S-Parameters of the back-to-back connected launcher, (**c**) front view of electrical vector distribution on the in-side launcher, and (**d**) side view electrical vector distribution on in-side launcher.

We now refer to the specified frequency response of the launcher in the frequency bandwidth of 8–20 GHz in the back-to-back form. Figure 6 illustrates the back-to-back configuration of the proposed launcher. We construct an error function as:

22 $$error \, function = \sum\limits_{k = 1}^{M} {\omega_{1} \left[ {10 - \left| {S_{11}^{dB} \left( {f_{k} } \right)} \right|} \right]^{2} + \omega_{2} \left[ {3 - \left| {S_{21}^{dB} \left( {f_{k} } \right)} \right|} \right]^{2} } \, , \, M = \frac{{f_{H} - f_{L} }}{frequncy \; step \; size}$$

where $\omega_{1} ,\omega_{2}$ are the weight factors and adjust the importance of each error function term, which are considered 2 and 1 in the design process, respectively. Also, *f*_*H*_ and *f*_*L*_ are upper and lower cut off frequencies of the operation bandwidth of the launcher that are assumed 8 and 10 GHz, respectively. The frequency step size is considered 10 MHz. The minimization of the error function using the genetic algorithm (GA) in the CST software gives the optimum dimensions of the launcher. The GA considered here as a global minimum seeking algorithm does not need initial values for variable. The optimized values of physical dimensions of the proposed launcher are listed in Table 2.

**Table 2:** Optimized dimensions of the proposed launcher.

Parameter	Value (mm)	Parameter	Value (mm)
W_1_	60	h_2_	1.5
W_2_	58	h_3_	0.5
L_1_	4	H_1_	16
L_2_	4	H_2_	2
L_3_	4	W_strip_	35
h_1_	3	L_T_	24

Figure 6b shows the simulated scattering parameters of the back-to-back design of the launcher. The simulated magnitude of S11 indicates a good impedance matching in the entire range of 8–20 GHz with an impedance bandwidth of about 142%. The simulated magnitude of S_21_ is above − 3 dB, indicating a good transmission performance. Figure 6c shows the electric field distribution at the *xoz* plane. Figure 6d also depicts how the electric field is distributed in the yoz plane inside the launcher. Figure 6d indicate that the proposed launcher creates a suitable modal matching between the feed and SSPP metaradiator.

### Implementation, full-wave simulation, and fabrication

It is challenging to choose the appropriate geometrical values for a unit cell that fully satisfies the impedance distribution of the hologram. This problem is further complicated in anisotropic unit cells, where the dimensions must simultaneously map to corresponding three values (*Z*_*xx*_, *Z*_*yy*_, and *Z*_*xy*_) for each unit cell of the hologram. Therefore, this paper uses the PSO algorithm, a fast convergence and efficient global search algorithm, to simplify the mapping process. The optimal geometrical parameters are retrieved by this algorithm, i.e., *a*, *b*, and $\psi$ of each unit cell. Three anticipated input entries of impedance matrix are thus considered the input parameters of PSO. In this way, the unit cell recognizes three predicted dimensions corresponding to the surface impedance matrix for that position while minimizing the below cost function at 18 GHz.

23 $$CF\_2 = \left| {Z_{xx} - Z_{xx\_desirable} } \right|^{2} + \left| {Z_{xy} - Z_{xy\_desirable} } \right|^{2} + \left| {Z_{yy} - Z_{yy\_desirable} } \right|^{2}$$

where $Z_{xx\_desirable} ,Z_{xy\_desirable}$ and $Z_{yy\_desirable}$ have been determined with (4), (5) and (6) formula.

**Fig. 7:**
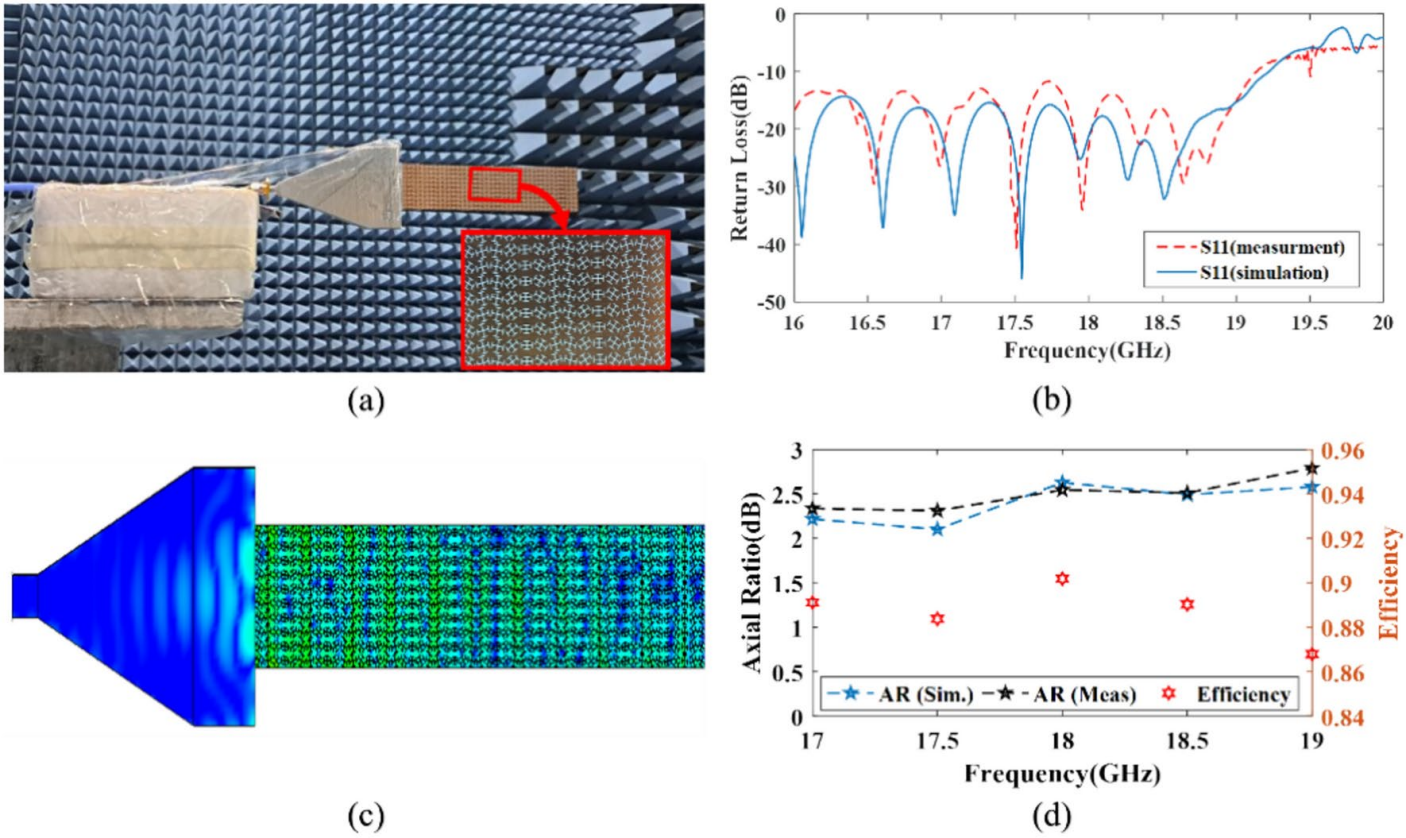
(**a**) Measurement environment of the antenna (the enlarged part shows a top zoom-in view of the proposed manufactured SSPP LWA), (**b**) simulated and measured reflection coefficient of the proposed SSPP LWA, (**c**) near electric-field distribution, and (**d**) simulated and measured AR as well as simulated radiation efficiency of SSPP LWA.

The whole structure of the hologram can be simulated using the full-wave simulation platform CST Studio Suite after extracting the ideal values of *a*, *b*, and $\psi$ employing the PSO. Figure 7a shows the fabricated proposed SSPP LWA structure based on an anisotropic holographic technique tested in an anechoic chamber room. The length of the hologram surface is $8 {\lambda }_{0}$, which consists of 400 anisotropic unit cells. Figure 7b shows the simulation and measurement results for S11 which are in good agreement. As can be seen, both simulated and measured S11 of the SSPP LWA are below − 10 dB within the frequency range of 17–19 GHz. As depicted in Fig. 7c, near electric-field distribution in the xoy-plane of SSPP LWA at 18 GHz is presented. According to Fig. 7d, the calculated radiation efficiency is higher than 93%, which agrees well with the full-wave simulation outcome, and the axial ratio (AR) is less than − 3 dB from 17 to 19 GHz. The results of the measured parameters are in good agreement with those of the simulated ones, which shows the quality of the design method.

**Fig. 8:**
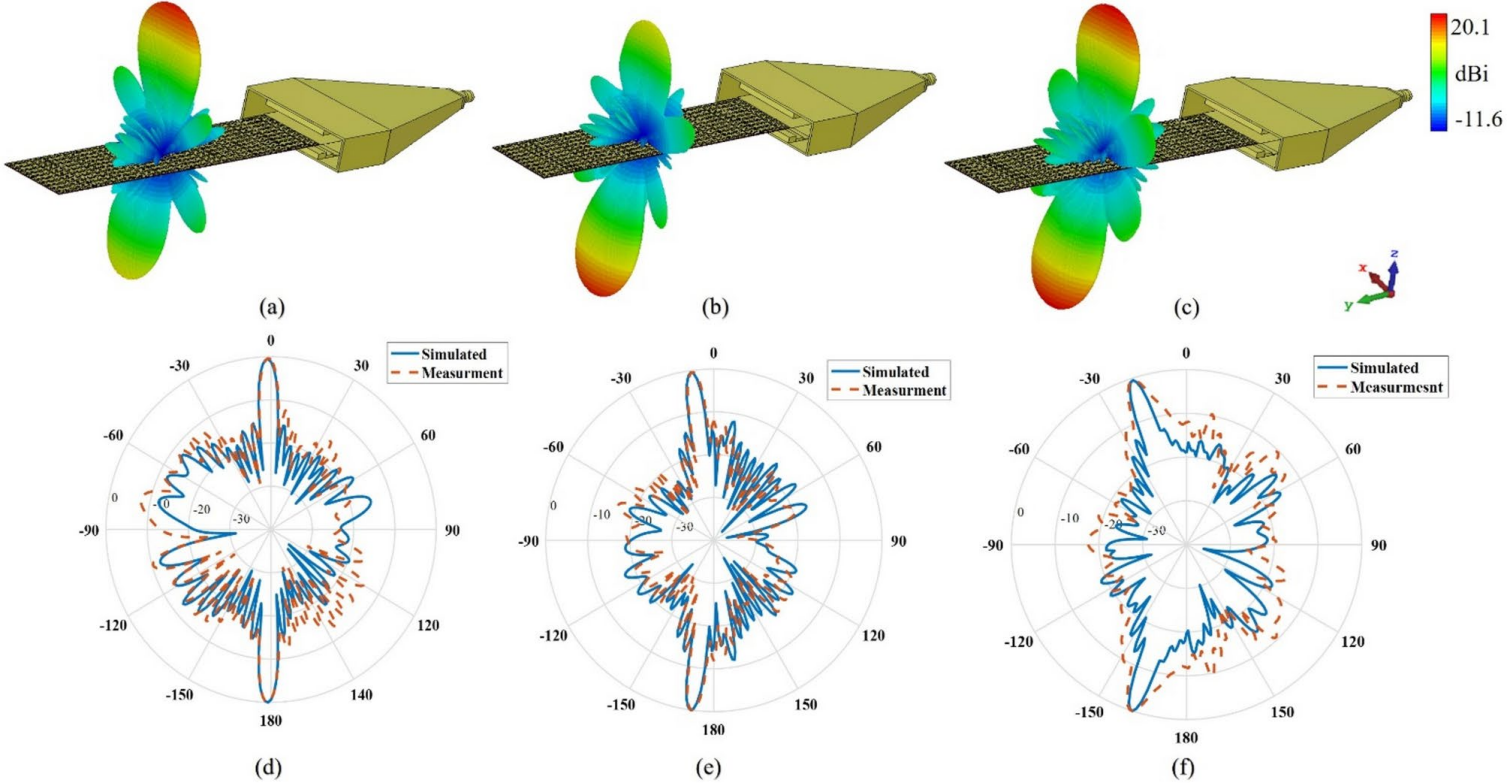
Simulated 3-D far-field radiation patterns at 18 GHz. (**a**) RHCP, (**b**) LHCP, (**c**) Absolute normalized measured and simulated polar patterns of $\varphi =\pi /2$ at (**d**) 16 GHz, (**e**) 18 GHz, and (**f**) 19 GHz.

Figures 8a–c show the 3D RHCP, LHCP, and absolute far-field radiation pattern of the SSPP LWA, respectively. In the proposed structure, the upper beam is RHCP, and the lower beam is LHCP. Figures 8d–f indicates the $\varphi =\pi /2$ plane polar radiation pattern of the proposed SSPP LWA, whose main beam can scan over a range of 18° by changing frequency from 17 to 19 GHz. With increasing frequency, the upper beam scans from 0° to 18° and the lower beam scans from − 180° to − 162°. The measurement and simulated SLL of the SSPP LWA are − 12.1 and − 13 dB, respectively.

The proposed structure is compared with the state-of-the-art SSPP LWAs using no parasitic elements in terms of realized gain at the design frequency, dimension, scanning range, radiation efficiency, and SLL in Table 3. The presented antenna has a realized gain of 19.7 dBi; meanwhile, as reported in^20^, it is about 12.1 dBi. Compared to other developed antennas, the radiation efficiency of the proposed antenna is more significant. For instance, we have attained 93%, while the antenna presented in^20^ has achieved almost 86% radiation efficiency. Furthermore, the proposed structure has smaller electrical dimensions and SLL compared to previous works.

**Table 3:** Comparison of the proposed SSPP LWA with the state-of-the-art studies.

References	f_0_ (GHz)	Unit cell	Polarization	Aperture length	Aperture width	Peak gain (dBi)	SLL	Scanning range	Rad. efficiency (%)	AR
^11^	9.3	Isotropic	Linear	$15\text{.}9{\lambda }_{g}$	$0\text{.}31{\lambda }_{g}$	12.2	− 9 dB	20°	90	–
^12^	9	Isotropic	Circular	$18\text{.}36{\lambda }_{g}$	$0\text{.}81{\lambda }_{g}$	12	− 10 dB	20°	77	13.9%
^13^	10	Isotropic	Linear & Circular	$24\text{.}9{\lambda }_{g}$	$0\text{.}25{\lambda }_{g}$	9	− 9 dB & − 6 dB	10°	80	4%
^27^	18	Anisotropic	Circular	$11\text{.3}9{\lambda }_{g}$	$0\text{.}65{\lambda }_{g}$	11.3	− 10 dB	20°	86	40%
This work	18	Anisotropic	Circular	$10\text{.}72{\lambda }_{g}$	$2\text{.}67{\lambda }_{g}$	19.7	− 12.1 dB	18°	93	11.1%

Moreover, according to Table 3, the aperture width of the proposed antenna is larger compared to other structures. A wider aperture results in a narrower the half power beamwidth (HPBW) of the H-plane pattern, altering the shape of the radiation beam from a fan-beam to a pencil-beam. Also, this increase in aperture width causes to promote the realized gain. In other similar launchers, the feed networks have limitations to uniform illumination of the radiation aperture with an arbitrary width. However, the proposed feed network can excite the radiation aperture with any desired width, uniformly without any destructive effects on the polarization purity of the far field radiation pattern.

## Conclusion

An SSPP LWA with circular polarization based on the anisotropic holographic technique fed by a novel launcher is designed, simulated, and tested. The anisotropic unit cell controls the main beam’s polarization without using parasitic patches. The design process involves using AFE, the holographic principle, and a combination of the TO equation and PSO algorithm. Also, a novel launcher excites SSPP waves with a small destructive effect on the radiation patterns. The beams on the upper and lower sides of the proposed LWA support RHCP and LHCP, respectively. According to the measurement results, the maximum scanning angle, peak gain, and SLL are 18°, 19.7 dBi, and − 12.1 dB, respectively. The radiation efficiency, beam scanning, and compactness aperture structure of this LWA improved compared to previous studies, making the proposed SSPP LWA as a good candidate for satellite wireless communication applications.

## Data Availability

Data sets generated during the current study are available from the corresponding author on reasonable request. Reprints and permissions information is available at www.nature.com/reprints.
